# Associations between prenatal diabetes mellitus, social determinants of health, and postpartum psychopathology: A retrospective review within women with diabetes mellitus

**DOI:** 10.21203/rs.3.rs-7032300/v1

**Published:** 2025-08-06

**Authors:** Allie Sidwell, Quetzal A. Class

**Affiliations:** University of Illinois College of Medicine; University of Illinois at Chicago

**Keywords:** Gestational diabetes, perinatal mental health, postpartum depression, type 1 diabetes mellitus, type 2 diabetes mellitus

## Abstract

**Background:**

We examine associations between pregestational Type 1 or Type 2 diabetes mellitus (PGDM) versus gestational diabetes mellitus (GDM) with postpartum psychopathology.

**Methods:**

We collected demographics, social determinants of health, medical comorbidities, psychiatric history, and postpartum psychiatric diagnoses at an urban, academic hospital from 9/2020-12/2023. Outcomes across patients with GDM and PGDM were compared with chi-square, t-tests, and multivariable logistic regression.

**Results:**

Of 6,186 pregnancies, 111 (3%) experienced PGDM and 871 (23%) GDM. Patients with PGDM showed higher rates of cesarean delivery [X^2^ (1) = 9.30, p < 0.01], NICU stay [X^2^(1) = 21.65, p < 0.0001], and MICU stay [X^2^(1) = 7.05, p < 0.01] and higher maximum HbA1c values [7.14 +/− 1.67 mmol/mol versus 6.18 +/− 1.49 mmol/mol; t(304) = 9.92, p < 0.01] as compared to those with GDM. Low social connections, a measured social determinant of health, was higher for patients with GDM (X^2^ (1) = 6.97, p < 0.01) as compared to PGDM. Women with PGDM were at double the odds of postpartum psychopathology compared to those with GDM after controlling for several measured covariates (OR = 2.40, 95% CI 1.55–3.73). However, when HbA1c was included in the model, the elevated risk was eliminated (OR = 1.03, 95% CI 0.35–3.04).

**Conclusion:**

As compared to GDM, PGDM was associated with increased odds of medical and social comorbidities. The relation between PGDM and postpartum psychopathology may be partially explained by elevated HbA1c.

## Introduction

Diabetes mellitus (DM) is a prevalent metabolic disease that places added stress on many body systems, including the cardiovascular, renal, nervous, and gastrointestinal systems.^1^ DM can manifest as Type 1 (T1DM), Type 2 (T2DM), or Gestational DM (GDM), distinctions that are driven by etiology and the timing of manifestation of symptoms. About 1-2% of pregnant people have T1DM or T2DM, and 6-9% of women develop GDM during pregnancy.^2^ Developing GDM increases a patient’s chance of developing T2DM after pregnancy with a lifetime risk of up to 60%.^3^ Furthermore, the percentage of pregnant people with GDM has more than doubled from 2000 to 2010, and during that same time period, the percentage of women with T1DM or T2DM increased by 37%, indicating that DM during pregnancy is a growing concern.^2^

Previous research indicates that psychopathology, such as major depressive disorder, is more than three times higher in individuals with T1DM and twice as high in those with T2DM as compared with the general population.^4,5^ Others have shown that quality of life is lower and depression/anxiety symptoms are high in nearly half of their studied GDM sample.^6^ Less is known, however, about specific differential risk across T1DM, T2DM, and GDM and postpartum psychopathology. Besides duration of disease, one biological difference across DM type may be glycated hemoglobin (HbA1c) levels, a common diagnostic measure of DM.^7^ Literature shows evidence of an association between HbA1c level and the development of depression.^8-10^ Additionally, early HbA1c levels have been shown to differentially predict diagnosis of GDM, the likelihood of fetal anomaly, perinatal death, and maternal preeclampsia.^11^

Perinatal mood disturbance is multifaceted with potentially long-term, far-reaching consequences for the mother and baby.^12^ Thus, we aim to elucidate the association between DM, pregnancy, and peripartum mood disorders. Specifically, we compare rates of peripartum psychiatric diagnoses across women with differing forms of DM while controlling for other important factors, such as social determinants of health and comorbid medical diagnoses.

## Methods

We conducted a retrospective cross-sectional study collecting data from electronic medical records of women who had a live birth at 22 gestational weeks or greater between March 1, 2020 and December 31, 2023. Initial data extraction from the University of Illinois Hospital in Chicago was performed by the Center for Clinical and Translational Sciences services following Institutional Review Board approval and waiver of informed consent (#2022 – 1045). We only included women diagnosed with GDM, T1DM or T2DM via ICD 9 and 10 codes (GDM: O24., T1DM: E10., T2DM: E11.). T1DM and T2DM were grouped into pre-existing DM (PGDM) categories. Each case was reviewed by hand (A.S.) for verification of diagnosis type and timing of prior to or within the current pregnancy.

Demographic characteristics and medical comorbidities collected included preeclampsia, hypertension, anemia, and vitamin D deficiency. We also included if the patient was taking insulin or metformin during the prenatal period and her glycated hemoglobin (HbA1c) values assessed during pregnancy. For analyses, we focused on the maximum HbA1c value collected during the pregnancy. In addition, blood glucose levels from the glucose stress test during pregnancy were collected for women with GDM. We also recorded birth and delivery factors as well as social determinants of health. Social determinants of health included alcohol use, nicotine use, degree of stress, depression, experiencing interpersonal violence, physical activity, social connections, transportation, financial, food security, and housing instability. These factors are asked frequently, if not at every prenatal visit. If the indicator was ever endorsed by the patient within the study time frame, it was considered as endorsed for study. Maternal historic diagnosis of a mental health disorder within the 5 years prior to delivery was also collected.

Primary outcomes included the scores of postpartum 2-week and 6-week Patient Health Questionnaire 9-question (PHQ9), as well as any perinatal diagnosis of depression, anxiety, and other psychopathologies within 1 year following delivery.^13^ These diagnoses were broad and included ICD 9: 648.42, 293.89, 684.44, 300.00, 293.1, 300.02, 300.3, 309.28, 296.99, 296.80, 296.10, 296.13, 296.14, 300.4, 296.2, 799.29, 296.30 and ICD 10: O90.6, O99.345, F53, O99.34, F41.9, F05, F41.1, F42.9, F43.20, R45, F31, F30, F34.1, F32.9, R45.89, F33.9.

### Planned analyses

Following descriptive analysis and univariate Chi-Square comparing PGDM and GDM across all measured factors, we used multivariable logistic regression to predict the categorical outcome of the presence of postpartum psychopathological diagnosis within a year of delivery. In a parallel fashion, we separately predicted PHQ9 depression screener scores from 2- and 6-week postpartum visits using Poisson regression to handle the count data of the depression screener.

Two regression models were performed with the main predictor being type of DM with GDM as the reference category for PGDM. The first model adjusted for gestational age at birth, type of delivery (cesarean or vaginal), and neonatal intensive care unit (NICU) admission. The second model additionally adjusted for HbA1c max level during pregnancy. Although insulin use, hypertension, preeclampsia, SDoH low social connectedness, and psychiatric diagnosis within the 5 years prior to the first prenatal visit were associated with DM type, they were also associated with postpartum psychiatric diagnosis outcome and therefore we did not include these potential colliders in the models.

**Figure 1:**
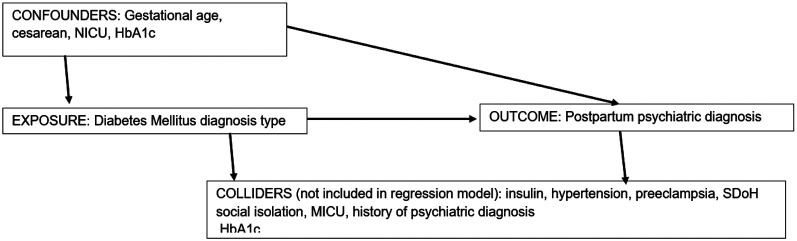
Directed acyclic graph outlining regression model Directed acyclic graph aided in the determination of variables included in the logistic regression model predicting postpartum psychopathology from diabetes type experienced during pregnancy. Colliders were removed from the model due to associations with the exposure and the outcome. Confounders were only associated with the exposure.

In Fig. 1 we present a directed acyclic graph (DAG). This illustrates the potential relation of exposure of DM type (pregestational or gestational), the confounders of gestational age, cesarean section, NICU admission, and maximum HbA1c, and the outcome of postpartum psychiatric diagnosis as well as a list of the potential colliders (hypertension, preeclampsia, insulin, MICU, low social connectedness social determinant of health, and prior psychiatric diagnosis) that we did not include in the models.^13^

## Results

Our sample began with 3,704 live births at 22 or more weeks gestation. When limiting the sample to women with a diagnosis of DM, 982 (26.5%) births remained. This included 871 with GDM diagnosed in the current pregnancy and 111 with PGDM made up of 54 T1DM and 57 T2DM diagnosed prior to the current pregnancy.

As presented in Table 1, univariate analyses showed that race, ethnicity, and insurance status did not differ across those with GDM versus those with PGDM. Those with PGDM, as compared to those diagnosed with GDM, were found to have higher rates of cesarean section deliveries [X^2^ (1, N) = 9.30, p < 0.01], NICU stays when limited to full-term births only [X^2^ (1, N) = 21.6524, p < 0.0001], MICU stays [X^2^ (1, N) = 7.051, p < 0.008], preeclampsia [X^2^ (1, N) = 3.9235, p < 0.0476], and hypertension [X^2^ (1, N) = 9.5190, p < 0.0020]. Gestational age at birth was shown to be significantly lower for women with PGDM (36.40+-3.70 weeks) than women with GDM [37.75 +-2.38 weeks; t(978) = 5.20, p < 0.001]. A higher than expected number of people with GDM endorsed SDoH factor social isolation as compared to those with PGDM, [X^2^ (1, N) = 6.97, p < 0.01]. For the SDoH factors of alcohol use, housing instability, intimate partner violence, and transportation difficulties, there were not enough cases of endorsement for these factors to be analysed. No differences across DM diagnosis type were noted across any other social determinants of health factors (Table 1).

Also presented in Table 1, DM-related medications and HbA1c showed differential rates between DM groups. In particular, we found that women were prescribed insulin [X^2^ (1, N) = 85.47, p < 0.01] and metformin [X^2^ (1, N) = 6.98, p < 0.01] more often if they had PGDM as compared to GDM. The maximum HbA1c levels measured at any point in pregnancy were also significantly higher in women with PGDM [7.14 (+/− 1.67) mmol/mol, range: 4.80–12.70 mmol/mol] as compared to GDM [6.18 (+/− 1.49) mmol/mol; range: 4.50–14.40 mmol/mol]. This was true when HbA1c was measured as a continuous values, [t(304) = 9.92, p = 0.0018 (p < 0.01)], and also when examined categorically with an above or below 6.5 mmol/mol binary indicator [X^2^(1) = 9.15, p < 0.0025]. HbA1c was not associated with postpartum psychiatric diagnosis [t(303) = 1.06, p = 0.29].

Women with PGDM showed higher rates of history of psychiatric diagnoses within the 5 years prior to their index pregnancy [X^2^ (1, N) = 24.97, p < 0.0001] as compared to women with GDM. As compared to women with GDM, univariate analyses showed that women with PGDM also had higher rates of postpartum or pregnancy-related psychiatric diagnosis within a year post-delivery, [X^2^ (1, N) = 21.94, p < 0.0001], one of our primary outcome variables. However, no significant differences in PHQ9 screening scores at 2- or 6-weeks postpartum were noted across diagnosis groups and therefore these outcomes were not studied using multivariable regression.

We then performed multivariable logistic regression predicting diagnosis of a psychiatric condition that adjusted for factors found to be significantly different across the DM groups plus core demographic characteristics. Results for the multivariable logistic regressions are presented in Table 2. In Model 1, which adjusted for gestational age, type of delivery, NICU, women with PGDM were at 2.40 higher odds (95% CI 1.55–3.73) of developing a postpartum psychiatric diagnosis within the first year postpartum as compared to women with GDM. In Model 2 we additionally adjusted for HbA1c in a subsample with HbA1c levels measured during pregnancy. With this additional adjustment, the elevated odds for postpartum psychiatric diagnosis for women with PGDM as compared with GDM was eliminated, [OR = 1.03 (95% CI 0.35–3.04)].

## Discussion

In our urban sample of mostly Black and publicly-insured patients, we found that a diagnosis of PGDM, meaning either T1DM or T2DM prior to the pregnancy, was associated with double the odds of pregnancy and postpartum-related psychiatric diagnoses in the first year postpartum when compared to women who received a new GDM diagnosis. This association was independent of several important measured covariates. Our findings support previous research showing postpartum PHQ9 scores were higher for patients with PGDM as compared to GDM.^15^ The seemingly time-limited nature of GDM may lessen the psychological impact of the new diagnosis as compared with the chronic nature of a PGDM diagnosis. Given that GDM often leads to a T2DM diagnosis, however, early screening and management of GDM is warranted, emphasizing the importance of integrated, longitudinal care.^3^

There are few other studies directly comparing GDM and PGDM, but rather comparing patients with one form of DM with patients without DM. For example, one study found that pregnant people with T1DM had significantly lower Standardized Mental Component scores than pregnant individuals without T1DM.^16^ While a prospective cohort study of 800 patients found that the Edinburgh Postnatal Depression Scale and Perceived Stress Scale did not differ between pregnant people with and without T1DM, though again this was not comparing within women with metabolic disorders.^17^ Additionally, postpartum mood is studied using numerous methods of measurement or other psychiatric illness and thus, associations vary naturally. When examining risk for psychological outcomes and T2DM, a case-control study found that T2DM during pregnancy was not a significant risk factor for psychopathology during pregnancy,^18^ but a prospective cohort study of 178 patients found that women with T2DM during pregnancy were significantly more likely to have symptoms of anxiety and/or depression.^5^ Meta analyses have shown both statistically significant relations between GDM and postpartum depression as well as no statistically significant relationship.^19, 20, 21^

Interestingly and importantly, however, in a smaller subcohort of our sample we found that when HbA1c was included in the predictive model, the association between diabetes type and postpartum psychological outcome was no longer significant. Our finding may help explain the high levels of variability in outcomes across this area of study. Our findings may also be interpreted as underscoring the important relation between blood glucose and mental health — an association that may transcend diagnostic categories. Previous work has presented biological mechanisms that support an association between blood glucose and metabolic function and mental health.^8-10^ Additionally, the walkability of one's community has been shown to lead to better glycemic control in women with pregestational diabetes, a factor that may be associated with several aspects of what are considered social determinants of health.^22^

Examining social determinants of health factors showed that women with GDM were at higher risk of endorsing social isolation as compared with women with PGDM. People with low social connection are more likely to delay seeking medical care.^23^ Additionally, social isolation and loneliness are linked to increased mortality^24^ and studies using social capital and neighborhood deprivation as markers for low social connection saw significantly increased rates of GDM.^25, 26^ However, increased rates of T2DM are also associated with social isolation and loneliness.^27, 28^ We encourage future research on this important factor.

While our study moves the field forward by comparing metabolically similar patients and including a breadth of measured covariates, there are important limitations to consider. First, many PHQ9 scores were not recorded within the patient electronic medical record. We are unsure if this was due to zero scores and therefore the provider did not enter a score or if the standard screener was not given to the patient. Second, at our hospital, the Edinburgh Postnatal Depression Scale may be used as an alternative to the PHQ9.^13^ There were a total of 34 2-week and 17 6-week Edinburgh Postnatal Depression Scores in our sample though none of these scores were recorded for women without PHQ9 scores. Therefore, we did not utilize the Edinburgh Postnatal Depression Scale information. Third, it may have been the case that while delivery occurred at our hospital, the patient received psychological care elsewhere and their diagnosis is not reflected in our data. In addition, previous work has suggested associations between antipsychotic medication and risk for GDM.^19^ While the confounding here may be limited due to the decreased likelihood of taking an antipsychotic prescription during pregnancy, future research should consider investigating the role of including psychiatric medications. Finally, though DM type and diagnosis was reviewed by hand, there are still inherent human errors in electronic medical records that are translated into study data.

Early and continuous screening for pregnancy and postpartum mental health disorders are warranted to prevent adverse events in the mother and child. Targeted interventions may be beneficial in certain populations including those with PGDM. Psychological interventions that specifically address the needs of pregnant and postpartum women with DM, perhaps that additionally target behavioral health changes in nutrition and glycemic control, may be beneficial for the management of both DM and mental health issues.

## Conclusions

In conclusion, individuals with PGDM were at higher risk for postpartum psychiatric diagnosis, as well as several adverse birth outcomes and comorbidities, as compared to GDM. The relation between diabetes type and postpartum psychiatric diagnosis was fully attenuated with the inclusion of HbA1c. Our study shows important differences between DM type experienced during pregnancy which may be important to consider in patient care. More research into the biological link between DM and mental health is warranted as HbA1c levels are manageable.

## Figures and Tables

**Table 1:** Demographic and background factors across diabetes mellitus diagnosis groups of pregestational diabetes mellitus (PGDM) or gestational diabetes mellitus (GDM)

Factors	GDM, n (% or sd)	PGDM, n (% or sd)	t or X^2^, df, p
n	111	879	—
BMI (kg/m^2^)	35.34, 863 (8.71)	37.07, 109 (9.80)	−1.92, 970, 0.055
Maternal age at delivery (yrs)	31.42, 871 (5.87)	32.54, 111 (5.74)	−1.89, 980, 0.059
Gestational age (weeks)	37.75, 869 (2.38)	36.40, 111 (3.70)	**5.20, 978, < 0.001**
Neonatal birth weight (g)	3121.52, 856 (677.98)	2983.25, 109 (880.06)	1.93, 963, 0.054
Postpartum 2-week PHQ9	2.10, 150 (3.311)	3.00, 5 (4.80)	−0.59, 153, 0.556
Postpartum 6-week PHQ9	2.59, 9 (3.754)	4.78, 9 (5.63)	−1.66, 182, 0.098
Race			11.06, 5, 0.0503
White	105 (12.06)	18 (16.22)	
Black	372 (42.71)	52 (46.85)	
Other	394 (45.23)	41 (36.93)	
Ethnicity			2.84, 3, 0.4164
Non-Hispanic	489 (56.14)	61 (54.95)	
Hispanic	318 (36.51)	39 (35.14)	
Declined or Unknown	64 (7.35)	11 (9.91)	
Insurance			0.07, 1, 0.7907
Private	222 (25.49)	27 (24.32)	
Public	649 (74.51)	84 (75.68)	
Cesarean Section			**9.30, 1, 0.0023**
No	493 (56.73)	46 (41.44)	
Yes	376 (43.27)	65 (58.56)	
NICU stay, full-term only			**21.65, 1, < 0.0001**
No	455 (56.73)	33 (32.35)	
Yes	347 (43.27)	69 (67.65)	
Maternal ICU Admission			**7.05, 1, 0.008**
No	684 (88.37)	74 (78.72)	
Yes	90 (11.63)	20 (21.27)	
Preeclampsia			**3.92, 1, 0.0476**
No	613 (70.46)	68 (61.26)	
Yes	257 (29.54)	43 (38.74)	
Hypertension			**9.52, 1, 0.0020**
No	433 (49.77)	38 (34.23)	
Yes	437 (50.23)	73 (65.77)	
Postpartum or Pregnancy-related Psychiatric Diagnosis within 1 year after delivery			**21.94, 1, < 0.0001**
No	684 (78.62)	65 (58.56)	
Yes	186 (21.38)	46 (41.44)	
SDoH Factors			
Depression			1.40, 1, 0.2471
No	811 (93.11)	100 (90.09)	
Yes	60 (6.89)	11 (9.91)	
Financial Struggles			1.54, 1, 0.2146
No	855 (98.16)	107 (96.40)	
Yes	16 (1.84)	4 (3.60)	
Food Instability			2.83, 1, 0.0925
No	863 (99.08)	108 (97.30)	
Yes	8 (0.92)	3 (2.70)	
Insufficient Physical Activity			2.69, 1, 0.1008
No	733 (84.16)	100 (90.09)	
Yes	138 (15.84)	11 (9.91)	
Social Isolation			**6.97, 1, 0.0083**
No	864 (99.20)	107 (96.40)	
Yes	7 (0.80)	4 (3.60)	
Stress			0.13, 1, 0.7214
No	845 (97.01)	107 (96.40)	
Yes	26 (2.99)	4 (3.60)	
Tobacco Use			1.63, 1, 0.2027
No	746 (85.65)	90 (81.08)	
Yes	125 (14.35)	21 (18.92)	
Alcohol			–
No	852 (97.82)	110 (99.10)	
Yes	19 (2.18)	1 (0.90)	
Housing Instability			–
No	869 (99.77)	111 (100.00)	
Yes	2 (0.23)	0 (0.00)	
Intimate Partner Violence			–
No	871 (100.00)	111 (100.00)	
Yes	0 (0.00)	0 (0.00)	
Transportation Issues			–
No	868 (99.66)	111 (100.00)	
Yes	3 (0.34)	0 (0.00)	
Insulin			**85.47, 1, 0.001**
No	589 (67.62)	25 (22.52)	
Yes	282 (32.38)	86 (77.48)	
Metformin			**6.98, 1, 0.0082**
No	547 (65.90)	59 (53.15)	
Yes	297 (34.10)	52 (46.85)	
HbA1c 6.5 or greater			**9.15, 1, 0.0025**
No	202 (72.40)	12 (44.44)	
Yes	77 (27.60)	15 (55.56)	

NOTE: SDoH: Social determinants of health ever endorsed within the study window

**Table 2:** Multivariable logistic regression models predicting pregnancy and postpartum psychiatric diagnosis in the year following delivery

Factors	Model 1, OR (95% CI), n = 901	Model 2, OR (95% CI), n = 272 subsample
PGDM (GDM reference)	2.40 (1.55–3.73)	1.03 (0.35–3.04)
Gestational age, weeks	0.95 (0.90–1.01)	1.00 (0.89–1.12)
Cesarean Section	1.00 (0.72–1.39)	0.89 (0.49–1.59)
NICU	1.12 (0.79–1.59)	1.19 (0.62–2.28)
HbA1c		0.92 (0.75–1.12)

## Data Availability

The data are available from the corresponding author upon reasonable request.

## Abbreviations

DM: diabetes mellitus
GDM: gestational diabetes mellitus
HbA1c: glycated hemoglobin
NICU: neonatal intensive care unit
PGDM: pregestational diabetes mellitus
PHQ9: Patient Health Questionnaire 9-question
T1DM: type 1 diabetes mellitus
T2DM: type 2 diabetes mellitus
